# Peri-lead edema and local field potential correlation in post-surgery subthalamic nucleus deep brain stimulation patients

**DOI:** 10.3389/fnhum.2022.950434

**Published:** 2022-09-08

**Authors:** Marco Prenassi, Linda Borellini, Tommaso Bocci, Elisa Scola, Sergio Barbieri, Alberto Priori, Roberta Ferrucci, Filippo Cogiamanian, Marco Locatelli, Paolo Rampini, Maurizio Vergari, Stefano Pastore, Bianca Datola, Sara Marceglia

**Affiliations:** ^1^Fondazione IRCCS Ca’ Granda Ospedale Maggiore Policlinico, Milano, Italy; ^2^Department of Engineering and Architecture, Università degli Studi di Trieste, Trieste, Italy; ^3^“Aldo Ravelli” Research Center for Neurotechnology and Experimental Brain Therapeutics, Department of Health Sciences, University of Milan Medical School, Milan, Italy; ^4^Neuroradiology Unit, Department of Radiology, Careggi University Hospital, Florence, Italy; ^5^Department of Pathophysiology and Transplantation, University of Milan, Milan, Italy

**Keywords:** DBS (deep brain stimulation), STN-DBS, brain laterality, DBS surgery, MRI

## Abstract

Implanting deep brain stimulation (DBS) electrodes in patients with Parkinson’s disease often results in the appearance of a non-infectious, delayed-onset edema that disappears over time. However, the time window between the DBS electrode and DBS stimulating device implant is often used to record local field potentials (LFPs) which are used both to better understand basal ganglia pathophysiology and to improve DBS therapy. In this work, we investigated whether the presence of post-surgery edema correlates with the quality of LFP recordings in eight patients with advanced Parkinson’s disease implanted with subthalamic DBS electrodes. The magnetic resonance scans of the brain after 8.5 ± 1.5 days from the implantation surgery were segmented and the peri-electrode edema volume was calculated for both brain hemispheres. We found a correlation (*ρ* = −0.81, *p* < 0.0218, Spearman’s correlation coefficient) between left side local field potentials of the low beta band (11–20 Hz) and the edema volume of the same side. No other significant differences between the hemispheres were found. Despite the limited sample size, our results suggest that the effect on LFPs may be related to the edema localization, thus indicating a mechanism involving brain networks instead of a simple change in the electrode-tissue interface.

## Introduction

Deep brain stimulation (DBS) implant consists of a subcutaneous electrical pulse generator (IPG) connected to electrodes surgically implanted into a target structure in the brain. DBS provides electrical neuromodulation to the target area that depends on the treated pathology (Krack et al., 2017). Many patients with advanced stage Parkinson’s disease (PD) benefit from DBS, specifically with implants in the subthalamic nucleus (STN), and is presently a common and well-established practice for the treatment of this disease (Volkmann et al., 2001; Deuschl et al., 2006; Castrioto et al., 2011; Krack et al., 2017). Stereotactic neurosurgery is required to precisely insert electrode leads which have to cross the brain cortex to reach the basal ganglia. After surgery, recent evidence suggests that a non-infectious, delayed-onset cerebral edema could appear around DBS electrode leads. This post-operative edema could be an asymptomatic (Englot et al., 2011; Fernández-Pajarín et al., 2017) or symptomatic (Deogaonkar et al., 2011; Lefaucheur et al., 2013) complication and is detected mostly through Computerized Tomography (CT) and Magnetic Resonance Imaging (MRI) performed at different times after the surgery.

DBS electrodes also allow to record local field potential (LFP) signals generated by the neurons in the target area, collecting useful physiological information for understanding deep brain pathophysiology and to optimize DBS therapy through closed-loop strategies that adapt stimulation parameters according to LFP changes (Kühn et al., 2006; Giannicola et al., 2010). LFPs are recorded in the time window between the surgery for electrode placement and that for IPG connection (usually 3–7 days; Arlotti et al., 2018, 2019). This implies that most of the research conducted so far is based on LFPs recorded in a so-called “acute” phase in which the electrode-tissue interface is not fully stabilized, also partly due to the presence of the peri-electrode edema. It has been long debated whether the results obtained in this phase are generalizable or not to a more chronic condition, when the electrode-tissue interface is more stable (Rosa et al., 2011; Giannicola et al., 2012); but in the fixed timeframe of this experiment (5–11 days after the surgery), it is expected a stable electrical environment for every patient in the areas near the electrode (Rosa et al., 2010).

Post-operative MRI is a useful tool to verify the correct electrodes’ placement and in the last 4 years, it has become a routine clinical practice for many patients undergoing DBS surgery. The purpose of this work is to study post-surgery edema and how it affects deep brain electrical activity, integrating the volumetric and spatial information gathered through post-surgery imaging (MRI scans), and the LFP signals recorded through the implanted electrodes.

## Methods

### Subjects

We enrolled eight rigid-akinetic patients with advanced PD undergoing surgery for subthalamic nucleus DBS electrode implantation at the Neurosurgery Unit at Fondazione IRCCS Ca’ Granda Ospedale Maggiore Policlinico in Milan from June 2017 to May 2018 without experiencing any surgical complication. The study was approved by the institutional review board and conformed with the Declaration of Helsinki, and all patients provided written informed consent to the experimental procedures.

All patients did not experience any surgical complications.

### Surgical procedure

DBS bilateral intracerebral lead implantation was performed under local anesthesia in awake patients, after 12 h withdrawal of anti-Parkinsonian drugs.

The STN target and trajectory were planned using CT-MRI fused images with a digitized stereotactic atlas (Borellini et al., 2019). Intraoperative monitoring procedures were applied to check the correct positioning of the leads during surgery (Marceglia et al., 2010). According to standard procedures, two exploratory microelectrodes recording brain electrical activity and able to functionally stimulate the target area were inserted to allow clinical assessment.

All patients were implanted in both hemispheres with macro-electrodes for DBS (Leads model 3389 Medtronic, Minneapolis, MN, USA). Each electrode has four cylindrical platinum iridium alloy contacts (1.27 mm in diameter, 1.5 mm in length, placed 2 mm apart, center-to-center) denominated in order 0–1–2–3 with 0 as the more caudal contact.

The targeting procedure follows the recommendations of the Italian DBS Study group (Marceglia et al., 2010), and post-implant stimulation with the final electrode was performed to check that the lead was positioned with the STN functional target.

To allow LFP recordings during the post-surgery session, the electrode leads were externalized before the implant of the subcutaneous IPG, which was implanted a few days later (up to seven).

### MRI acquisition

Informed consent to MRI scans was obtained from all patients. MRI scans were carried out between 7 and 11 days after the implantation surgery (median 8.5 ± 1.5 days, see Table 1).

**Table 1:** Patient data.

**Patient**	**Sex**	**PD onset side**	**Handedness**	**Time between surgery and the MRI scan**
P1	F	Right	Right	9
P2	F	Left	Right	9
P3	M	Left	Right	11
P4	M	Left	Right	8
P5	M	Left	Right	8
P6	M	Right	Right	7
P7	F	Left	Right	8
P8	M	Left	Left	9

The post-operative MRI scans were performed with a Philips Achieva 1.5 T system, with a maximum gradient slew rate per axis less than or equal to 200 T/m/s and with a maximum spatial field gradient less than or equal to 40 T/m. Scan sequence throughout the scan had average head SAR (specific absorption rate) up to 0.1 W/kg or less. During every MRI scan axial TSE T2, sagittal 3D-TFE, T1 and 3D-FLAIR images with axial and coronal reconstruction were collected; diffusion weighted images (DWI) were also included and DWI and Apparent Diffusion Coefficient (ADC) maps were calculated. The volume of the T2-hyperintensity surrounding each DBS lead was quantified by one radiologist using a manual volume segmentation method.

A minor T2 hyperintensity was present near the leads in every sequence due to magnetic susceptibility artifact. To take this into account, an MRI was considered positive for edema if the T2 hyperintensity measured at least 0.7 ml.

**Figure 1:**
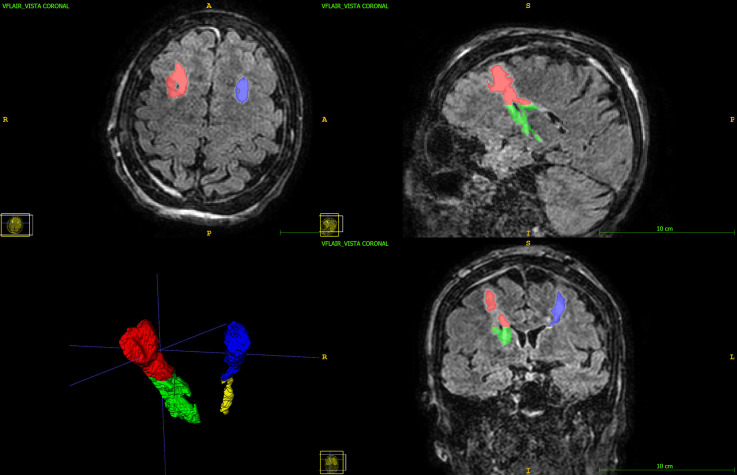
3D FLAIR image with multiplanar reconstruction in axial, coronal, and sagittal planes. The subcortical edema (red and blue) and the basal edema (green and yellow) Volumes of Interest are displayed.

FLAIR images were chosen for segmentation as they allow the best contrast for edema visualization that appears as a T2 hyperintense area. Additionally, FLAIR sequences are 3D sequences with isotropic voxels and, therefore, they allow the segmentation of a Volume of Interest. ITK-SNAP software allows the segmentation of Volumes Of Interest (VOI) and 3D image measurements. The edema area was manually segmented obtaining Regions of Interest (ROIs) on several contiguous slices of a series on FLAIR images (Figure 1). Next, the ROIs were then interpolated using the tool “interpolate labels” and considering the spacing between slices. This gives correct measurements of VOI. The VOI including all the T2-hyperintense areas corresponding to edema was further segmented on FLAIR images in subcortical edema and basal edema. The boundary between the two regions was arbitrary chosen as an axial plane at the level of the roof of the cella media of lateral ventricles as this corresponds to the cranial boundaries of basal ganglia and capsular system (e.g., internal capsule) and at the same time is easy to identify and well reproducible. The segmentation was performed for the right and left sides separately obtaining the subcortical and basal edema volumes for eachs side.

The FLAIR images were volumetric 3D sagittal images with multiplanar reconstruction in axial and coronal planes with an isotropic voxel size of 1 × 1 × 1 mm = 1 mm^3^.

### Local field potentials recordings

In the morning of the 5th day after surgery and 12 h after withdrawal of anti-Parkinsonian medication, LFPs were recorded with the patient at rest from electrode contact pairs 0–2, 0–3, and 1–3. Signals were acquired using a biomedical signal amplifier (gain 80 dB, passband 1–100 Hz, notch ON; Model Grass ICP511, Astromed, USA) and then digitalized at 256 samples/s with 16-bit quantization *via* an ADC Micro1401-3 unit (Cambridge Electronic Design, UK) connected to a PC. The signals were analyzed using the Spike 2 software (Cambridge Electronic Design, UK) and the Matlab Software (version 9.3.0.713579, R2017b; The MathWorks, Natick, USA). The signal acquisition duration was at least 30 s but never exceeded 60 s.

A Welch’s power spectral estimate with a Hamming window of 256 samples (1 s), 50% of overlapping, and spectral resolution of 1 Hz was calculated for every signal.

For the analysis, we chose the signal coming from the contact pair showing the highest peak in the beta band (10–35 Hz). This was done because the signal with the most significant peak is considered a good control variable of the motor condition of the patient (Giannicola et al., 2010).

**Figure 2:**
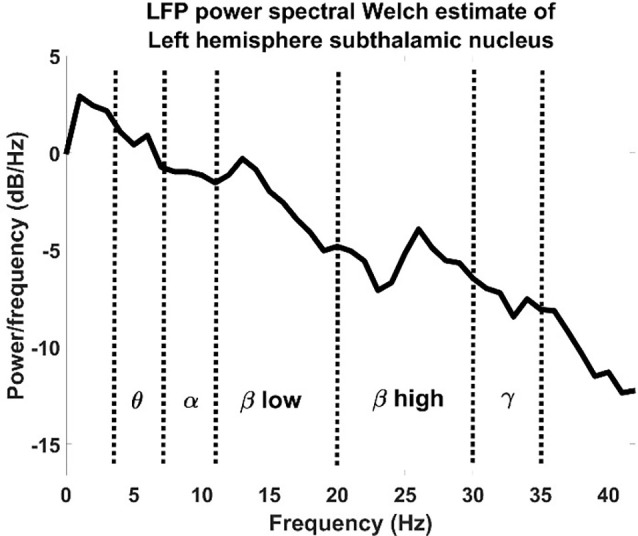
LFP power spectral Welch estimate of an LFP recorded from a left hemisphere subthalamic nucleus divided by physiological bands.

The resulting spectra were normalized using z-score normalization shown in Equation (1) and then divided by bins relative to the physiological bands (Figure 2): theta band: 5–8 Hz, alpha band: 8–11 Hz, low beta band: 11–20 Hz, high beta band: 20–30 Hz, gamma bands: 30–35 Hz.


(1) $$ZWsignal_{p}=\frac{pWsignal_{p}-\bar{pWsignal_{p}}}{\sigma \left(pWsignal_{p}\right)}$$

Where p: patient, *pWsignal*: power spectral density estimate calculated applying the Welch method, $\bar{pWsignal}$: power spectral density average, and σ: standard deviation.

The statistical significance was set to *p* < 0.025, resulting from the Bonferroni correction of two, due to the double analysis of effects (effect of the laterality and effect of the edema localization) of a classical *p* < 0.05.

## Results

### Patient status

The patients (three females, five males) had a mean age of 57 ± 7 years old, with a mean disease duration of 13 ± 3 years. The perioperative course was unremarkable in all patients but one, a 64-year-old male who had a short and mild confusional state in the first 10 days after the operation. At 1 year follow up our patients’ outcome was in line with most of the previously published studies. Mean OFF medications UPDRS variation after one-year follow up was a 27% reduction. Quality of life means improvement was 28% measured with a PDQ39 variation.

### Edema volume

The segmented volumes are reported in Table 2. A Wilcoxon rank sum test on the data confirms that the left and right hemispheres are not statistically different (*p* > 0.6), with a mean of 8.57 cm^3^ and a standard deviation of 5.08 cm^3^ for the right side and a mean of 6.55 cm^3^ and a standard deviation of 5.13 cm^3^. The total basal edema average is 2.31 ± 2.03 cm^3^, and the total subcortical edema volume average is 5.25 ± 3.85 cm^3^. The larger areas interested by the edema are located in the subcortical regions between the prefrontal subcortex and the premotor area. These regions present lateralized characteristics (Riecker et al., 2002; Lemaire et al., 2013).

**Table 2:** MRI edema volume.

	**Total Edema volume**		**Basal Edema volume**		**Subcortical Edema volume**	
**Patient**	**right hemisphere**	**left hemisphere**	**right hemisphere**	**left hemisphere**	**right hemisphere**	**left hemisphere**
	**[cm^3^]**	**[cm^3^]**	**[cm^3^]**	**[cm^3^]**	**[cm^3^]**	**[cm^3^]**
P1	18.69	12.29	6.09	2.15	12.60	10.14
P2	6.92	3.29	3.43	0.83	3.49	2.46
P3	2.85	0.73	1.88	0.62	0.97	0.11
P4	11.82	11.95	7.62	3.60	4.20	8.35
P5	4.84	11.84	0.841	2.38	4.00	9.46
P6	10.21	0	1.41	0	8.80	0
P7	4.62	7.83	1.69	1.70	2.93	6.13
P8	8.57	4.45	0.96	1.73	7.61	2.72
**Mean**	8.57	6.55	2.99	1.63	5.57	4.92
**St. dev.**	5.08	5.13	2.55	1.14	3.79	4.13

St.dev, standard deviation.

The differences in volume between the right and left basal sub-volumes are not statistically significant (right side basal sub-volume mean: 2.99 ± 2.55 vs. left side basal sub-volume mean 1.63 ± 1.14, *p* = 0.2, Wilcoxon’s sign-rank test).

Left PD onset for this relatively small sample is prevalent, with only two right side cases.

### Local field potentials analysis

A scalar sum of each band (theta, alpha, low, and high beta and gamma) of the right and left STN sides was calculated separately as shown by the Equation (2).


(2) $$BandPower_{p,b}=\sum_{n=frange\_\min}^{frange\_\max}ZW{\mathrm{signal}}_{p}$$

Where p: patient, b: frequency band, frange_min, and frange_max: minimum and maximum frequency range in Hz (see Table 3, frequency band and frequency range); ZWsignal: pwelch power spectrum density estimate.

**Table 3:** Local field potentials left and right comparison.

**Frequency band**	**Frequency range**	**Mean Band Power [dB/H_Z_]**				**Wilcoxon probability left vs. right hemisphere**
		**Mean Left**	**St.Dev. Left**	**Mean Right**	**St.Dev. Right**	
Theta	5–8	−88.04	11.60	−92.10	17.44	0.51
Alpha	8–11	−83.35	15.96	−86.45	20.26	0.88
Low beta	11–20	−77.06	17.45	−78.51	18.75	0.38
High beta	20–30	−86.21	14.48	−88.70	18.36	0.33

h = 0: same distribution.

The table left and right STN LFP signal did not show a statistically significant inter-subjective difference between any of the five frequency bands analyzed, as reported in Table 3 (Wilcoxon rank sum test). Except for the onset side, these results show that there is no difference between the left and right sides if we analyze MRI scans and LFP signals separately.

### Edema volumes and local field potentials

We correlated each left and right band power (z-score as calculated in Equation 2) for each patient with the corresponding edema volume.

**Figure 3:**
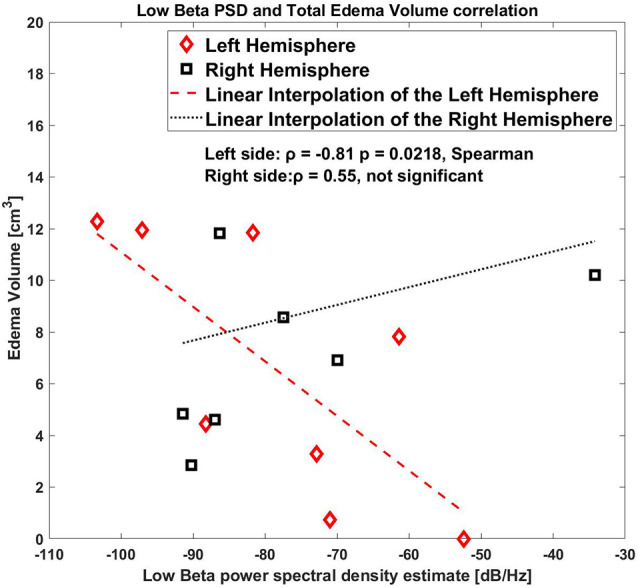
Correlation between low beta band power and the edema volume for both sides, right and left hemispheres.

When considering left and right sides together, there was not a statistically significant correlation, [ρ = −0.74 *p* = 0.042, Spearman, (1-β) = 0.6 in low beta band]. Figure 3 shows a representative example of the correlation between these two variables for the low beta band including both hemispheres.

As shown by the correlation plot (Figure 3) the datapoint on only the left side were clearly aligned where the right side was scattered.

**Figure 4:**
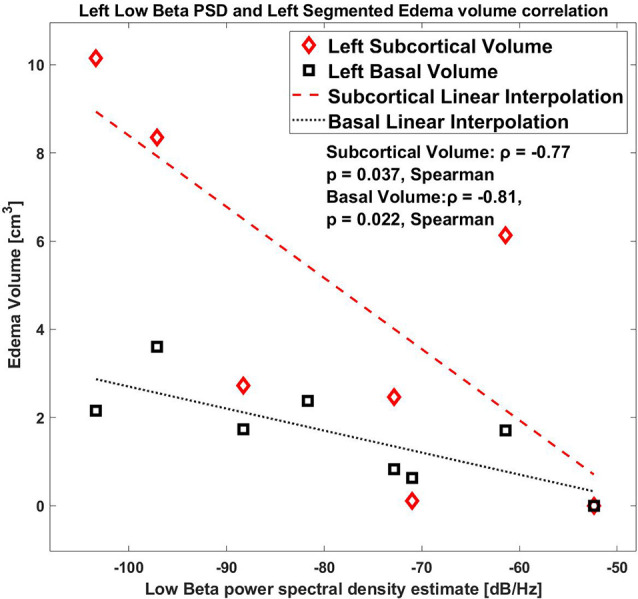
Correlation between low beta band power and the edema volume for the left hemisphere with Basal and Subcortical segmentation.

As a further analysis, we correlated the values for the right and the left hemisphere separately, and we found that the low beta band (10–20 Hz) for the left side STN only showed a significant correlation with the edema volume (ρ = −0.81, *p* = 0.0218, Spearman, (1-β) = 0.73, Figure 4), whereas this correlation was completely absent in the right side (ρ = 0.55, *p* = 0.171, (1-β) = 0.3).

Regarding the edema sub-volumes: the left low beta correlates with the left subcortical volume, ρ = −0.77 *p* = 0.037, Spearman; also, the left low beta correlates with the left peri-electrode volume ρ = −0.81 *p* = 0.0218, Spearman.

## Discussion

In this work the integration of two methodologies, MRI scan segmentation and LFP recordings are used to investigate the short-term post-surgery in STN-DBS. As the first results show, if the methodologies are used individually, they give a limited, even if still powerful, insight into the patient status, the surgery outcome, and the brain functionality.

In fact, taken separately, both the MRI scans and LFP recordings show no difference between the right and left hemispheres, by a large margin. Conversely, combining the two methodologies, we found a possible specific pattern of inverse correlation between the low beta LFP oscillation and the edema volume on the left hemisphere which is not present in the right hemisphere. This inverse correlation implies that, in the left hemisphere, the synchronicities of the low beta waves are decreasing as the edema size grows. This is an interesting result especially for PD, as the beta band is correlated with the motor condition of the patient.

Our results, therefore, suggest that there is a possible correlation between the presence of edema and the activity recorded, which may affect the interpretation of LFP results in the immediate post-surgical period. It is intuitive that the presence of peri-electrode edema may affect the electrode-tissue interface and, in turn, LFP power. However, our observations show that the correlation is specific to the LFP beta band, and not to the total LFP power. Moreover, the correlation is both with the peri-electrode edema and with the total edema, including the sub-cortical portion. Finally, the correlation we observed is localized in the left hemisphere, and it is not visible in the right one. Altogether, these results may suggest that the effect of edema is not only related to the electrode-tissue interface but other network mechanisms affecting information transmission in the basal ganglia thalamocortical loop may be involved in the beta power edema-related changes.

Even though the edema volume in sub-cortical areas is more than twice the volume of the peri-electrode edema, they both contribute to the correlation with beta power and we cannot determine which is the major cause of the edema-related effect. However, the sub-cortical regions interested by the edema are between the prefrontal cortex and the premotor area, and present lateralized characteristics. Therefore, the localization on the left side of the correlation deserves further investigation, since it cannot be simply explained by the clinical asymmetry of the disease (e.g., disease onset) or of the surgical procedure (e.g., different edema volume on the two sides).

A first hypothesis may refer to an inter-hemispheric asymmetry underlying the nigrostriatal degeneration (Obeso et al., 2004); in this scenario, a recent work has also suggested that lateralization of cortical beta activity likely reflects disease severity, whereas lateralization of cortical alpha activity may represent a potential marker of levodopa responsiveness (Mostile et al., 2015). Although no conclusive remark has been reached so far, a converging evidence suggests that the right hemisphere is more prone to neurodegeneration than the left one, as confirmed by higher dopamine levels found in the left compared to the right striatum (Wagner et al., 1983; Volkow et al., 1996a, b; Fuente-Fernández et al., 2000; Dyck et al., 2002; Haaxma et al., 2010). Nonetheless, sub-cortical recordings have failed to demonstrate differences in the beta power between the left and right STN.

A second possible explanation relies on the well-known predominance of the hyper-direct pathway of basal ganglia networks within the right hemisphere (Jahfari et al., 2011). This asymmetry may lead to a sustained inhibition of the right thalamocortical loop in PD patients, thus increasing the beta power and minimizing the effect of edema on LFP recordings when compared to the left STN.

Our study is limited by some aspects. The first limitation is the small sample size and the predominance of right-handed male individuals. Also, the position of the edema, not only in-depth but also in the subcortical area, is not investigated in this study. The results are statistically weakened by the various other tests that have been carried out in this preliminary study, and by the univariate nature of the analysis. However, the absence of significant correlation in the primary analysis (i.e., left plus right volume correlation with low beta LFP) may be in favor of the subsequent lateralized analysis. We cannot, however, completely rule out the possibility that the correlation we observed may represent a “false positive” discovery. Moreover, the edema volume in our sample, as assessed by MRI, is larger in the right than the left hemisphere in six out of eight patients, possibly adding a confounding factor. Finally, a distinction between hypokinetic and tremor-dominant forms of the disease may reveal some differences in the relationship between tissue edema and beta power. Widening the patient samples, especially including more patients with right PD onset, obtaining a finer spatial characterization of the edema, and including clinical assessments are the objectives of future studies.

## Conclusion

The combination of the MRI scans and the STN-LFP signals, even with a small sample size, highlighted a correlation between the normalized STN-LFP and post-surgery DBS peri-electrode edema in the low beta band in the left side hemisphere. This suggests that the presence of edema may affect LFP recordings, even though the mechanisms have also to be clarified in the future with further studies.

## Data Availability Statement

The raw data supporting the conclusions of this article will be made available by the authors, without undue reservation.

## Ethics Statement

The studies involving human participants were reviewed and approved and the study was approved by the institutional review board of Fondazione IRCCS Ca’ Granda Ospedale Maggiore Policlinico in Milan. The patients/participants provided their written informed consent to participate in this study.

## Author Contributions

SM and MP: conceptualization, investigation, formal analysis, supervision, data curation, writing—original draft, writing—review and editing. LB and TB: investigation, conceptualization, writing—original draft, writing—review and editing. AP: investigation, writing—review and editing. SP, RF, FC, PR, ML, and MV: conceptualization, investigation, and writing—review. BD: formal analysis and data curation. All authors contributed to the article and approved the submitted version.

## Funding

The present work was partially supported by the PAIN RELife project by Regione Lombardia (POR FESR 2014–2020/ Innovazione e competitività).
